# Parental death by external causes and risk of hospital-treated deliberate self-harm in bereaved offspring

**DOI:** 10.1007/s00787-020-01534-3

**Published:** 2020-04-21

**Authors:** Lisa Victoria Burrell, Lars Mehlum, Ping Qin

**Affiliations:** National Centre for Suicide Research and Prevention, University of Oslo, Sognsvannsveien 21, 0372 Oslo, Norway

**Keywords:** Deliberate self-harm, Parental bereavement, Suicide, Register study, Childhood, Adolescence

## Abstract

Previous studies have reported an increased risk of hospital-treated deliberate self-harm (DSH) in offspring who have lost a parent, but inconclusive findings regarding differences between loss from suicide and accidents. The present study aimed to investigate the association between parental death by external causes before age 18 and hospital-treated DSH, and potential differences between different accidents and suicide. This nested-case–control study was based on data from longitudinal Norwegian registers. Subjects comprised 12,526 people born between 1970 and 2003 who received acute somatic treatment because of DSH at hospitals and associated services between 2008 and 2013 (cases), and 222,362 controls matched for gender and date of birth with no recorded DSH treatment. Information concerning deceased parent’s death and offspring’s DSH treatment and socioeconomic data was merged. Data were analysed with conditional logistic regression. Results indicated that offspring who had lost a parent to suicide (OR 2.32, 95% CI 1.92–2.80) and death by accidents such as falls, poisoning, and drowning (OR 1.79, 95% CI 1.38–2.33) had a significantly increased risk of hospital-treated DSH compared to offspring who had not experienced such loss. Parental bereavement from transport accidents and other external causes were not associated with significantly increased risks. No differences were evident for different genders of deceased, ages at bereavement, or genders of bereaved. The improved identification of bereaved offspring at particular risk of hospital-treated DSH should be utilized to implement effective prevention and treatment programs in specialist healthcare aimed at the individuals at highest risk.

## Introduction

Approximately 30 million people experience the death of a close family member or friend to suicide, accidents, or homicide worldwide every year [1, 2]. Naturally, the children of the deceased are highly impacted by this loss given the broken attachment to a primary care giver [3] and the direct effect of loss on daily life [4]. The sudden death of a parent is consequently associated with an increased risk of long-term psychosocial sequela [5–7], and several Scandinavian population-based register studies have reported an increased risk of hospital treatment for deliberate self-harm (DSH) in bereaved offspring [5, 8–17]. When directly comparing offspring bereaved by suicide and accidents, researchers have reported an earlier onset of DSH hospitalization in offspring following parental suicide compared to those parentally bereaved by accidental death [9]. This higher risk of DSH hospitalization seems, however, to be linked to maternal, but not paternal suicide [8]. Studies comparing bereaved offspring to offspring of alive parents, however, fail to report a significant difference in DSH hospitalization between offspring bereaved by suicide and accidents [5, 17]. Since results are inconclusive regarding whether offspring bereaved by suicide are at a higher risk of hospital-treated DSH than those bereaved through accidents, further research is warranted. In particular, different accidental deaths may be associated with different risks of hospital-treated DSH in offspring due to the heterogeneity of accidents, potentially accounting for the inconsistencies in the previous studies. Further knowledge concerning the groups of bereaved offspring at highest risk may be helpful in tailoring postvention efforts and making them more targeted.

The present register study aims to investigate the association between parental death by external causes during childhood and adolescence and risk of hospital-treated DSH into adulthood. External causes of death refer to deaths where the cause is external to the body, such as accidents, suicides, and homicides, and is a classification in the ICD coding system. We aim to address the following research questions:

- To what extent are different external causes of death associated with an increased risk of hospital-treated DSH, and how do the risks associated with different external causes of death vary depending on other bereavement-related aspects?
- How does offspring’s risk of hospital-treated DSH vary depending on the gender of the deceased parent, the gender of the bereaved offspring, offspring’s age at bereavement, and the time since bereavement?

## Methods

### Data sources

The present nested-case–control study retrieved individual-level data from four longitudinal Norwegian registers and merged them by utilizing the personal identification number. The Central Population Register provided data on gender and residence address and contains a link to parent’s personal identification number, enabling identification of biological or adoptive parents. Statistics Norway’s Events Database provided information on ethnicity, marital status, education, income, and sick leave pension. Data concerning parental death were retrieved from the Cause of Death Register which contains the cause and date of all deaths in Norway coded according to ICD-8 (International Classification of Deceases, Eight Revision) from 1969 to 1985, ICD-9 from 1986 to 1995, and ICD-10 from 1996 to 2012 [18]. Finally, the Norwegian Patient Register provided data on hospital-treated DSH and the psychiatric diagnosis at the time of treatment. This register contains information on all contacts related to all types of treatments in specialist health care, covering all public institutions and private institutions and medical specialists contracted to the regional health authorities [19]. As a result of the Norwegian health care system’s universality and the register’s broad coverage, the register includes all cases of hospital-treated DSH in Norway since 2008, coded according to ICD-10. The study was approved by the Regional Committees for Medical and Health Research Ethics (ref. 2013/1620/REK South East) and owners of the relevant registers.

### Study design and population

In the present study, deliberate self-harm refers to intentional self-poisoning and self-injury, irrespective of suicidal intent [20, 21].

Cases were individuals born between 1970 and 2003 who received acute somatic treatment because of DSH at hospitals and associated services (i.e., specialist healthcare services), and who were hence recorded in the Norwegian Patient Register (NPR), between January 1st 2008 and December 31st 2013. We considered only cases who were at least 10 years old at the time of DSH treatment and excluded all indirect contacts, planned treatments, fatal injuries, and poisonings or injuries that were clearly accidental, inflicted by others or secondary outcomes of other medical conditions. Due to underreporting of DSH in the Scandinavian health registers [22, 23], we adopted a broader approach to include episodes of probable DSH to prevent detection bias. Based on the previous register-based research [22] and our examination of data on determined incidents which were given a diagnosis of DSH (ICD-codes X6n, Y87), three steps were followed in a hierarchical fashion to ascertain probable DSH episodes. The first step was to include 4421 cases with treatment contacts because of injuries with a comorbid diagnosis of DSH (ICD-codes X6n, Y87). The second step was to include 3966 cases with treatment contacts that had a diagnosis of either poisoning (ICD-codes T4n, T50–T55, T57–T60, T62, T65, and T96) or injuries (ICD-codes S10–S11, S15, S19, S21, S25–S27, S31, S35–S39, S41, S45, S50–S51, S54–S56, S59, S61, S64–S65, S69, S71, S88, T01, T09, T11, T18–T19, T27–T28, T31, T68, T69, T71 and T95), and had a comorbid diagnosis of mental or behavioural problems (ICD-codes F0–F9). The final step was to include 4139 cases with treatment contacts for poisoning (ICD-codes T4n, T50, and T96) that were not covered by the previous steps. The first recorded contact by a person was used as the case contact, resulting in 12,526 cases with DSH treated in specialist healthcare services during the study period. We will use the term DSH hospitalization to denote these registered hospital contacts.

A nested-case–control design [24] was then applied to select up to 20 controls for each case with no recorded history of DSH hospitalization in the study period. Controls were matched for date of birth, gender, and the date of DSH hospitalization, and were drawn from a 25% random sample of the population registered in the Central Population Register. This procedure resulted in 222,362 matched controls. Only individuals with a registered link to both their father and mother were included in the study.

### Variables of interest

The explanatory variable of interest is exposure to parental death by external causes (parental DBEC) before age 18 (ICD-8 and ICD-9 codes E800–E999; ICD-10 codes V01–Y89). Subjects were classified into categories of *bereavement status* as (a) no exposure to parental DBEC, or b) exposure to parental DBEC. Specific c*auses of parental death* were classified as a) suicide (ICD-8 and ICD-9: E950–E959, ICD-10: X60–X84), (b) transport accidents (ICD-8: E800–E845, ICD-9: E800–E848, ICD-10: V01–V99), (c) other accidents (ICD-8: E850–E929, ICD-9: E849–E869 and E880–E928, ICD-10: W00–X59), and (d) other external causes (ICD-8: E930–E949 and E960–E999, ICD-9: E870–E879, E929–E949 and E960–E999, ICD-10: X85–Y89). The low number of homicides in Norway makes separate analyses impossible, and homicides were hence included in the “other external causes” category. *Gender of deceased parent* was further classified as (a) father, (b) mother, and (c) both parents. Furthermore, subjects were classified according to their *age at bereavement* into (a) ≤ 4 years, (b) 5–9 years, (c) 10–14 years, and (d) 15–18 years. Finally, *time since bereavement* refers to the time between parental DBEC and DSH hospitalization or matching, and subjects were classified as (a) up to 5 years, (b) 5–9 years, and (c) 10–15 years. If both parents died, bereavement-related variables were classified according to the parent who died first.

Several covariates were included in the multivariate analyses. *Ethnicity* was classified as (a) born in Norway with two Norwegian born parents, (b) immigrant, (c) born in Norway with immigrant parents or one-parent born abroad, and (d) born abroad with one or two Norwegian born parents. *Highest education* at the time of DSH hospitalization or matching was classified as (a) no education or unknown education, (b) compulsory education, (c) high school and vocational education, and (d) higher education (University or College). *Taxable income* the year before DSH hospitalization or matching was calculated based on the Norwegian National Insurance Scheme’s basic amount G which is adjusted for annual national wage inflation [25]. Taxable income was classified as (a) up to 2G, (b) 2–4G, (c) 4G and more, and (d) unknown. *Marital status* at DSH hospitalization or matching was classified as (a) married, (b) never married, (c) separated, (d) divorced, (e) widowed, and (f) unknown. *Marital stability* and *residence stability* refer to the number of changes in marital status and residence address, respectively, and were classified as (a) 0, (b) 1, and (c) 2 or more. The *Number of sick leave episodes* the last 3 years before DSH hospitalization or matching was classified as (a) 0, (b) 1, (c) 2, (d) 3–4, and (e) 5 or more. *Diagnosis of sick leave episode* the last 3 years before DSH hospitalization or matching was classified as (a) no sick leave, (b) depression, (c) other psychiatric conditions, (d) musculoskeletal conditions, and (e) other.

### Statistical analyses

The outcome variable of interest was DSH hospitalization, and risk was estimated by a conditional logistic regression analysis [26]. Odds ratios (ORs) and 95% confidence intervals (95% CI) were estimated, and the analyses were conducted using IBM SPSS Statistics, version 25 (IBM Corp., Armonk, NY). Regression models were analysed in multiple steps. First, univariate analyses yielded crude ORs controlled for age, gender, and time of DSH hospitalization through matching. Second, multivariate analyses included all covariates to yield adjusted ORs. Finally, a hierarchical regression investigated the relative contribution of each covariate to identify the covariates with predictive power when statistically controlling for the effect of parental DBEC. The order of inclusion of covariates into the model was partially based on a related previous study by the authors [27], and was as follows: diagnosis of sick leave episode, number of sick leave episodes, marital status, taxable income, marital stability, highest education, residence stability, and ethnicity.

In the present study, 48.6% of cases received a primary or secondary psychiatric diagnosis at the time of DSH hospitalization. We performed a stratified multivariate regression for strata where the case had received a psychiatric diagnosis at DSH hospitalization and strata where the case had not received a diagnosis. Additionally, we performed a stratified multivariate regression for strata that were identified through the three different steps in the inclusion process.

Since study participants were born from 1970 and information about DSH hospitalization was available from 2008, participants may have had prior DSH events that were not included in the register. To investigate offspring’s first DSH hospitalization, we restricted the data set to people born from 1998 who hence turn 10 years when we have registered information in the NPR. For this subset of participants (186 cases and 3418 controls), we investigated bereavement status and time since bereavement as independent variables. We included only ethnicity and residence stability as covariates, since the young age of the participants rendered information on marital status, education, income, and sick leave irrelevant.

Furthermore, a sensitivity analysis for unobserved confounding [28] was implemented to adjust for the unobserved confounding from parents’ history of psychiatric hospitalization. The parameters which we selected were based on previous studies, and the odds ratio for the relationship between parents’ history of psychiatric hospitalization and offspring’s DSH hospitalization (OR_yu_) was set to 2.09 [12]. The prevalence of a history of psychiatric hospitalization in parents who died from suicide [*p*(*u*|*x* = 1)] was set to 0.45 [5], and the prevalence of a history of psychiatric hospitalization in the general population [*p*(*u*|*x* = 0)] was set to 0.06 [5]. We used these parameters to determine an adjustment factor, and the OR was adjusted for this factor to obtain the OR for the true association, free of bias from observed and unobserved confounding. Notably, the prevalence of psychiatric hospitalization in parents who died from suicide (45%) is markedly higher than the corresponding prevalence in parents who died from accidents (21%) [5], and was utilized in this analysis to yield the most conservative estimate of the adjusted OR.

Interactions between variables of study and gender of the bereaved, cause of death and gender of the deceased parent, and cause of death and the offspring’s age at bereavement were investigated using the log-likelihood ratio test. Interactions with gender of the bereaved were based on multivariate analyses, while interactions with cause of death were based on univariate analyses, since coefficients in the multivariate analyses did not converge.

## Results

Of the 12,526 people who had received treatment in specialized health care for DSH during the observation period, 56.4% were females. The mean age at DSH hospitalization was 24.3 years for females and 27.3 years for males. Overall, 287 cases and 2078 controls had experienced parental death by external causes before they turned 18 years. The distribution of the variable categories is presented in Table 1.

**Table 1:** Distribution (%) of the study variable categories among cases with hospital-treated deliberate self-harm and matched controls, for all subjects and males and females separately

	All subjects		Females		Males	
	(*N* = 234,888)		(*N* = 132,562)		(*N* = 102,326)	
	Cases	Controls	Cases	Controls	Cases	Controls
Variable	(12,526)	(222,362)	(7097)	(125,465)	(5429)	(96,897)
Bereavement status						
No exposure to parental DBEC	12,239 (97.7)	220,284 (99.1)	6931 (97.7)	124,330 (99.1)	5308 (97.8)	95,954 (99.0)
Exposure to parental DBEC	287 (2.3)	2078 (0.9)	166 2.3)	1135 (0.9)	121 (2.2)	943 (1.0)
Cause of death						
No exposure to parental DBEC	12,239 (97.7)	220,284 (99.1)	6931 (97.7)	124,330 (99.1)	5308 (97.8)	95,954 (99.0)
Suicide	148 (1.2)	871 (0.4)	88 (1.2)	468 (0.4)	60 (1.1)	403 (0.4)
Transport accidents	54 (0.4)	592 (0.3)	24 (0.3)	327 (0.3)	30 (0.6)	265 (0.3)
Other accidents	76 (0.6)	517 (0.2)	47 (0.7)	299 (0.2)	29 (0.5)	218 (0.2)
Other external causes	9 (0.1)	98 (0.05)	7 (0.1)	41 (0.03)	2 (0.04)	57 (0.1)
Gender of deceased						
No exposure to parental DBEC	12,239 (97.7)	220,284 (99.1)	6931 (97.7)	124,330 (99.1)	5308 (97.8)	95,954 (99.0)
Father	216 (1.7)	1637 (0.7)	121 (1.7)	898 (0.7)	95 (1.7)	739 (0.8)
Mother	63 (0.5)	412 (0.2)	39 (0.5)	215 (0.2)	24 (0.4)	197 (0.2)
Both parents	8 (0.1)	29 (0.01)	6 (0.1)	22 (0.02)	2 (0.04)	7 (0.01)
Age at bereavement						
No exposure to parental DBEC	12,239 (97.7)	220,284 (99.1)	6931 (97.7)	124,330 (99.1)	5308 (97.8)	95,954 (99.0)
≤ 4 years	67 (0.5)	509 (0.2)	32 (0.5)	279 (0.2)	35 (0.6)	230 (0.2)
5–9 years	74 (0.6)	544 (0.2)	47 (0.7)	292 (0.2)	27 (0.5)	252 (0.3)
10–14 years	83 (0.7)	627 (0.3)	53 (0.7)	349 (0.3)	30 (0.6)	278 (0.3)
15–18 years	63 (0.5)	398 (0.2)	34 (0.5)	215 (0.2)	29 (0.5)	183 (0.2)

Data are given as number (percentage) of each group. Percentages may not total 100 because of rounding

*DBEC* death by external causes

Table 2 displays the results from the univariate and multivariate regression analyses, for all subjects and for daughters and sons separately. Children and adolescents who had experienced parental death by external causes had a significantly increased risk of DSH hospitalization compared to offspring who had not experienced such loss (adjusted OR 1.83, 95% CI 1.60–2.10). The associated risks were somewhat reduced in the multivariate analyses after controlling for all covariates, and the hierarchical regression indicated that all covariates significantly predicted DSH hospitalization when the effect of parental DBEC was accounted for. After controlling for the unobserved confounding from parents’ history of psychiatric hospitalization, the risk of DSH hospitalization in offspring who had experienced parental DBEC remained significantly increased (OR 1.31, 95% CI 1.14–1.50).

**Table 2:** Odds ratios (95% confidence intervals) from the univariate and multivariate conditional logistic regression analyses indicating risk of hospital-treated deliberate self-harm associated with the variables under study, for all subjects and males and females separately. Log-likelihood ratio tests for gender differences

Variable	Crude OR^a^	Adjusted OR^b^			Interaction with gender^c^	
	All subjects	All subjects	Females	Males	*χ*^2^	*p*
Bereavement status					1.30	0.25
No exposure to parental DBEC	1 (reference)	1 (reference)	1 (reference)	1 (reference)		
Exposure to parental DBEC	2.49 (2.20–2.83)^‡^	1.83 (1.60–2.10)^‡^	1.98 (1.66–2.36)^‡^	1.69 (1.37–2.08)^‡^		
Cause of death					4.31	0.37
No exposure to parental DBEC	1 (reference)	1 (reference)	1 (reference)	1 (reference)		
Suicide	3.07 (2.58–3.66)^‡^	2.32 (1.92–2.80)^‡^	2.54 (1.99–3.24)^‡^	2.06 (1.53–2.78)^‡^		
Transport accidents	1.62 (1.23–2.15)^‡^	1.26 (0.93–1.69)	1.12 (0.72–1.73)	1.42 (0.94–2.15)		
Other accidents	2.69 (2.11–3.42)^‡^	1.79 (1.38–2.33)^‡^	1.97 (1.42–2.75)^‡^	1.63 (1.07–2.49)*		
Other external causes	1.66 (0.84–3.29)	1.13 (0.55–2.31)	1.72 (0.73–4.05)	0.54 (0.13–2.28)		
Gender of deceased					2.16	0.54
No exposure to parental DBEC	1 (reference)	1 (reference)	1 (reference)	1 (reference)		
Father	2.38 (2.06–2.74)^‡^	1.76 (1.51–2.06)^‡^	1.85 (1.51–2.26)^‡^	1.71 (1.35–2.16)^‡^		
Mother	2.78 (2.13–3.63)^‡^	2.00 (1.51–2.66)^‡^	2.37 (1.64–3.42)^‡^	1.58 (1.00–2.48)		
Both parents	5.12 (2.34–11.20)^‡^	3.09 (1.30–7.36)*	3.19 (1.19–8.53)*	2.82 (0.50–15.79)		
Age at bereavement					3.35	0.50
No exposure to parental DBEC	1 (reference)	1 (reference)	1 (reference)	1 (reference)		
≤ 4 years	2.38 (1.84–3.07)^‡^	1.69 (1.29–2.23)^‡^	1.64 (1.12–2.42)*	1.80 (1.22–2.68)^†^		
5–9 years	2.46 (1.93–3.14)^‡^	1.77 (1.37–2.30)^‡^	2.09 (1.50–2.91)^‡^	1.41 (0.92–2.16)		
10–14 years	2.37 (1.88–2.98)^‡^	1.81 (1.42–2.32)^‡^	2.02 (1.48–2.76)^‡^	1.53 (1.02–2.31)*		
15–18 years	2.90 (2.22–3.78)^‡^	2.14 (1.60–2.87)^‡^	2.18 (1.47–3.23)^‡^	2.15 (1.40–3.31)^‡^		

*DBEC* death by external causes

*p < 0.05, ^†^*p* < 0.01, ^‡^*p* < 0.001

^a^The ORs derived from these models were adjusted for age, gender, and calendar time through matching

^b^The ORs derived from these models were further adjusted for ethnicity, highest completed education, taxable income, marital status, marital stability, residence stability, number of sick leave pension episodes, and diagnosis of sick leave pension episode

^c^Interactions with gender of bereaved offspring were based on multivariate analyses

When performing a stratified multivariate regression for subjects who received a psychiatric diagnosis at DSH hospitalization and subjects who did not receive a diagnosis, no large differences were evident; OR 1.92, 95% CI 1.58–2.32 for DSH treatment with a psychiatric diagnosis, and OR 1.76, 95% CI 1.45–2.14 for DSH treatment without a psychiatric diagnosis. Similarly, no large differences were observed in the stratified analysis for the three DSH identification steps; OR 1.88, 95% CI 1.49–2.37 for step 1, OR 1.97, 95% CI 1.55–2.51 for step 2, and OR 1.87, 95% CI 1.46–2.39 for step 3.

The analysis of the first DSH hospitalization in subjects born after 1998 found that children and adolescents exposed to parental DBEC had close to eight times higher risk of DSH hospitalization compared to their non-exposed peers (OR 7.97, 95% CI 3.63–17.49). Markedly, only 10 cases and 21 controls had experienced parental DBEC in this subsample.

In the multivariate regression investigating cause of death, offspring exposed to parental suicide and death by other accidents showed a significantly increased risk of DSH hospitalization, while parental death by transport accidents and other external causes were not associated with a significantly increased risk (Table 2). The category of other accidents consisted of a large proportion of poisonings (39.0%), including overdoses from narcotics, medications and alcohol, and falls (16.2%). Parental suicide was associated with the highest odds ratio with more than a doubled risk of DSH hospitalization, and the non-overlapping confidence intervals shows that suicide is associated with a significantly higher risk of DSH hospitalization than transport accidents.

**Fig. 1:**
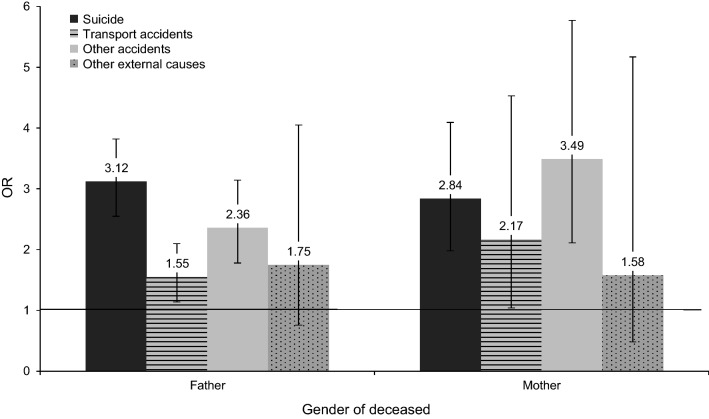
Odds ratios (95% confidence intervals) for the interaction between cause of death and the gender of the deceased parent. The horizontal line marks an OR of one

**Fig. 2:**
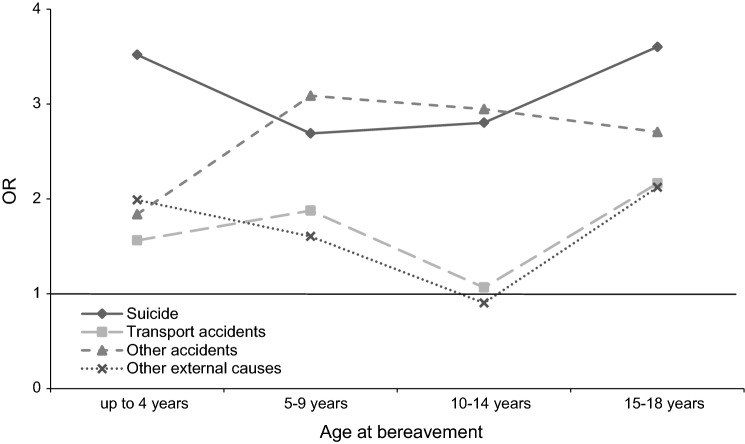
Odds ratios for the interaction between cause of death and offspring’s age at bereavement. The horizontal line marks an OR of one

The log-likelihood ratio test for the interaction between cause of death and gender of the deceased parent was non-significant (*χ*^2^: 4.99, *p*: 0.55), with parental suicide and death by other accidents being associated with the largest risks regardless of losing a mother or father (Fig. 1). Likewise, the log-likelihood ratio test for the interaction between cause of death and offspring’s age at bereavement was non-significant (*χ*^2^: 6.05, *p*: 0.74), with parental suicide and death by other accidents being associated with the greatest risks across all ages of bereavement (Fig. 2).

In the multivariate regression investigating gender of the deceased parent, paternal death, maternal death, and death of both parents were all associated with a significantly increased risk of DSH hospitalization in offspring (Table 2). Furthermore, no large differences between losing a father and mother were evident. The loss of both parents was associated with the highest risk of DSH hospitalization, but this group only consisted of 37 people.

When examining offspring’s age at bereavement, loss of a parent until 18 years was associated with a significantly increased risk of DSH hospitalization (Table 2). For offspring born after 1998 where the DSH hospitalization is the first episode, the investigation of time since bereavement found significantly increased risks of DSH hospitalization the first 10 years after bereavement (up to 5 years: OR 15.61, 95% CI 4.07–59.92; 5–9 years: OR 7.03, 95% CI 2.33–21.18), but no significantly increased risk 10–15 years after the loss (OR 3.49, 95% CI 0.40–30.24). Notably, the lower sample sizes in these subgroups are evident in the large confidence intervals.

The log-likelihood ratio tests investigating interactions between all bereavement-related variables and gender of the bereaved offspring were non-significant, indicating comparative associations between parental DBEC and DSH hospitalization in daughters and sons (Table 2).

## Discussion

In the present study, we found that children and adolescents who lost a parent to external causes of death had a significantly increased risk of hospital-treated DSH compared to offspring who had not experienced such loss. This increased risk can be attributed to parental suicide and death by accidents such as falls, poisoning, and drowning, and was evident in both daughters and sons. Offspring displayed an increased risk following bereavement throughout their childhood and adolescence and after loss of a mother, father, and both parents.

The present study is the first to discern the effects of transport accidents and other accidents on risk of DSH hospitalization in the bereaved offspring, and found different risks associated with different types of accidents. Previous register studies have reported an association between DSH hospitalization and parental bereavement from suicides and accidents in general [5, 17]. However, in the present study, offspring bereaved by parental death from suicide and accidents such as poisoning and falls had significantly increased risks, while deaths from transport accidents and other external causes were not associated with significantly increased risks. These results persisted irrespective of the gender of the deceased parent or the offspring’s age at bereavement. A misclassification of suicides as accidental deaths in the Norwegian Cause of Death Register is uncommon [29] and hence an unlikely explanation for the present findings.

Researchers have reported that the risk of mental disorder is significantly higher in people who die from accidental poisonings and falls than people who die in transport accidents [30]. The psychopathology related to accidental poisonings and falls will probably have influenced the family environment prior to the death of the parent, leading to an upbringing with insecurity, instability, and family discord. This may also be a reason for the increased risk of DSH hospitalization in offspring bereaved by parental suicide, given that a problematic family environment consisting of psychiatric disorders, maltreatment, abuse, and family discord is particularly present in families where members have died by suicide [31, 32]. Likewise, both suicide and accidental poisoning by medications may entail a period of physical illness preceding the death [33]. Genetic transmission of factors reported to highly influence suicidal behaviour, such as psychiatric disorders, neurocognitive deficits, and certain personality traits, may also explain the increased risk of DSH hospitalization following parental suicide and death from the other accidents [34]. Parental suicide was associated with the highest risk of DSH hospitalization in the present study, potentially due to grief specific variables related to suicide bereavement, stigma of suicide, feelings of responsibility, social isolation, anger at the deceased, and a ruminative need to understand the suicidal motivation [35–37].

The comparable risks of DSH hospitalization in offspring who have lost a mother and father are in accordance with the previous register-based studies investigating parental suicide [11] and death from all causes [13, 17]. Likewise, the lacking differences in risk between daughters and sons support previous findings [10, 11, 13, 15]. This lack of gender differences can potentially best be explained by the relative gender equality in Scandinavian countries with respect to child rearing responsibilities [38, 39], leading to comparable psychosocial impacts of maternal and paternal bereavement. Additionally, gender equality may yield both daughters and sons the ability to express their feelings, seek support, and process the bereavement. In accordance with previous studies [5, 12–14, 16], the present study found that bereavement throughout childhood and adolescence is associated with a significantly increased risk of DSH hospitalization. Developmental psychology has consistently stressed the importance of early experiences on later development, and adolescence has especially been conceptualized as a sensitive period, since experiencing traumatic events at this age can have a larger detrimental effect compared to exposure at other ages [40–42]. Furthermore, the first 5 years following bereavement were associated with the largest risks of DSH hospitalization, a finding similar to previous studies reporting the highest risk the first 2 years after bereavement [9, 10, 17]. The immediate effects following bereavement may be a direct effect of the trauma and loss itself, while the more long-term effects may be a result of developmental challenges related to growing up in a one-parent home and genetic heritability of suicidal behaviour and psychiatric disorder [34].

Results from the present study reporting an increased risk of DSH hospitalization in bereaved offspring are consistent with prior Scandinavian register studies [5, 10–15, 17], and add to the literature, suggesting that many bereaved offspring in Norway suffer from extensive psychosocial sequela [6, 7]. The explanation for the increased risk following bereavement may to a certain degree lie in the covariates included in the study, since the associations were attenuated in the multivariate analyses and all covariates significantly predicted DSH hospitalization. These interpersonal, intrapersonal, and health-related covariates may function as mediators in the association between parental bereavement and hospital-treated DSH. The interpersonal resources of being married and having stable residence and marital status may reflect social support and community network, and have previously been found to be protective against suicide in bereaved offspring [27]. Furthermore, reduced intrapersonal resources due to low income and education may entail reduced skills and life competence [43], further reducing capacities for coping and recovery. The covariates of greatest importance may be related to mental and physical health, reflected in the number of sick leave episodes and the diagnosis of the last sick leave episode, since the loss of an important attachment figure may cause or aggravate psychiatric disorders [36, 44–46]. In addition to the effect of these potential mediators, there seems to be a direct effect of bereavement on DSH, since the increased risk persisted after controlling for covariates and unobserved confounding. This direct effect may be because the sudden nature of external causes of death means that the bereaved have no or very little chance to prepare themselves or say goodbye, inducing a sense of loss of control. Moreover, the death is often accompanied by shock and strong sensory impressions, and may be experienced as a trauma. This in turn may lead to rumination and counterfactual thoughts about the death, in addition to the grief following bereavement in general.

For the study of low-frequency phenomena such as DSH hospitalization and parental DBEC, the present study has its strength in the use of national registers covering the entire population, enabling a large sample size and increased statistical power. Moreover, register studies do not suffer from problems with selection and attrition, nor biases related to observation or interviewing. Data in Norwegian registers are collected systematically and uniformly, and the data have been found to have high quality [19, 29, 47]. The registers are monitored, analysed, and corrected continuously [19, 48, 49]. Together, the strengths of the present study indicate that the study results have high external validity.

Results from the present study must be interpreted in light of some limitations. DSH is operationalized in a manner enabling the use of registers where the X6n deliberate self-harm code is insufficiently used. Because of this, we have widened the inclusion of DSH hospital treatment to include other ICD-codes, as explained in the Methods section. Therefore, some accidents may have been erroneously classified as deliberate self-harm, and actual acts of self-harm may have been mistakenly omitted. The sensitivity analysis stratifying by step of DSH identification, nevertheless, shows similar results. In community studies, cutting is the most common method of self-harm, while poisoning is the most common self-harm method in individuals presenting to hospitals [50]. Results from the present study consequently cannot be generalized to all the other self-harm studies. A further limitation relates to data access and the inability to include potential covariates, such as family socioeconomic status, whether the parents are the child’s primary caregivers, and the quality of the parent–child attachment. Finally, because the study excluded all offspring without a registered link to parents in the Central Population Register, our sample probably excluded disproportionately many first generation immigrants, since their parents are not registered in Norwegian registers.

In conclusion, children and adolescents bereaved by parental suicide and accidents such as poisoning and falls have a significantly increased risk of hospital-treated DSH. This increased risk is evident in both daughters and sons, following the loss of a mother, father or both parents. Clearly, the effects of bereavement on DSH hospitalization are extensive, and early exposure has long-lasting impacts.

Given the increased risk of DSH hospitalization in bereaved offspring, all bereaved offspring should be offered follow-up in primary healthcare and hence have the opportunity to seek help easily. For children and adolescents parentally bereaved through suicide, follow-up in specialized child and adolescent mental health services should additionally be offered on a routine basis. Wherever available, peer-group support specifically targeting families and youth bereaved through suicide should also be offered. We believe that it is important to use the data from this research to more effectively identify vulnerable groups and implement effective prevention and treatment programs in specialist healthcare aimed at the individuals at highest risk. Indeed, counselling and support groups for children and adolescents bereaved by suicide can be beneficial in reducing psychopathology [51]. The increased knowledge of high-risk groups should also be incorporated into risk assessments. Additionally, the school system can function as first responders to this vulnerable group through identifying and monitoring those at risk and offering support through school counselling services. The close association between schools and specialized care such as clinics for child and adolescent psychiatry and child protective services makes educators and school nurses well suited at referring bereaved children and adolescents for further follow-up. Identification of bereaved offspring at particular risk of psychosocial sequela is well underway, paving the way for more effective and specialized postvention.
